# Crinipellins A and I, Two Diterpenoids from the Basidiomycete Fungus *Crinipellis rhizomaticola*, as Potential Natural Fungicides

**DOI:** 10.3390/molecules23092377

**Published:** 2018-09-17

**Authors:** Jae Woo Han, Mira Oh, Yu Jeong Lee, Jaehyuk Choi, Gyung Ja Choi, Hun Kim

**Affiliations:** 1 Center for Eco-friendly New Materials, Korea Research Institute of Chemical Technology, Daejeon 34114, Korea; jaewoo82@krict.re.kr (J.W.H.); purunmei2002@naver.com (M.O.); 22yujeong@gmail.com (Y.J.L.); kjchoi@krict.re.kr (G.J.C.); 2 Department of Green Chemistry and Environmental Biotechnology, University of Science and Technology, Daejeon 34113, Korea; 3 Division of Life Sciences, Incheon National University, Incheon 22012, Korea; jaehyukc@incheon.ac.kr

**Keywords:** antifungal, *Crinipellis rhizomaticola*, crinipellin, biological control, mushroom

## Abstract

In the course of screening for microbes with antifungal activity, we found that the culture filtrate of the IUM00035 isolate exhibited strong antifungal activity against *Magnaporthe oryzae* and *Colletotrichum coccodes in planta*. Based on the phylogenetic analysis with the ITS region, the IUM00035 isolate was identified as *Crinipellis rhizomaticola*. To identify antifungal compounds from the *C. rhizomaticola* IUM00035 isolate, the culture filtrate of the isolate was partitioned with ethyl acetate and *n*-butanol and, consequently, two active compounds were isolated from the ethyl acetate extract. The chemical structures of the isolated compounds were determined as crinipellin A (**1**) and a new crinipellin derivative, crinipellin I (**2**), by NMR spectral analyses and a comparison of their NMR and MS data with those reported in the literature. Crinipellin A (**1**) exhibited a wide range of antifungal activity in vitro against *C. coccodes*, *M. oryzae*, *Botrytis cinerea*, and *Phytophthora infestans* (MICs = 1, 8, 31, and 31 µg/mL, respectively). Furthermore, when plants were treated with crinipellin A (**1**) (500 µg/mL) prior to inoculation with fungal pathogens, crinipellin A (**1**) exhibited disease control values of 88%, 65%, and 60% compared with non-treatment control against tomato late blight, pepper anthracnose, and wheat leaf rust, respectively. In contrast to crinipellin A (**1**), crinipellin I (**2**) showed weak or no activity (MICs > 250 µg/mL). Taken together, our results show that the *C. rhizomaticola* IUM00035 isolate suppresses the development of plant fungal diseases, in part through the production of crinipellin A (**1**).

## 1. Introduction

Plant-pathogenic fungi cause severe plant diseases, resulting in a loss of crop yields and economic damage to the agricultural industry [1]. Synthetic fungicides have been recognized as a primary method to control plant diseases caused by fungal pathogens, although the overuse of chemical pesticides has caused severe problems, such as toxicity for human health and environmental pollution. Therefore, the development of safer and more effective alternatives is necessary to replace chemical pesticides [2]. Among the various natural resources, antagonistic microbes (or microbial metabolites) have been recognized as an attractive source for new antifungal agents [3].

Mushrooms have been used as a functional food or as active ingredients of folk medicine due to their various bioactive compounds [4]. Currently, over 140,000 species of mushrooms have been reported, and more than 200 species have been known to possess antimicrobial properties [5,6]. The fruiting bodies, mycelial biomass, or culture filtrates of mushrooms contain bioactive compounds with a wide range of antimicrobial activity, including antifungal and antibacterial activities [7,8,9]. In particular, strobilurins, produced by wood-inhabiting forest mushroom *Strobilurus tenacellus* and *Oudemansiella mucida*, are one of the greatest findings in the history of fungicides [10]. Strobilurins act as inhibitors of the respiratory chain at the quinone outer binding site of the cytochrome *bc*_1_ complex [11]. Due to the unique and specific modes of action, a number of strobilurin analogues have been synthesized with improved antifungal activity, stability, and dissemination, of which several have been developed as highly successful commercial products such as kresoxim-methyl, pyraclostrobin, and trifloxystrobin [12].

In the search for antifungal agents from mushrooms, we recently found that a culture filtrate of *Crinipellis rhizomaticola* IUM00035 isolate has the potential to control plant diseases caused by *Magnaporthe oryzae* and *Colletotrichum coccodes*. Furthermore, we isolated and identified the active small molecules from the culture filtrate of *C. rhizomaticola* IUM00035 and investigated their in vitro and in vivo antifungal activity. Taken together, our results provide a useful information for the development of plant-protecting agents using microbial-derived materials to control plant diseases caused by fungal pathogens.

## 2. Results and Discussion

### 2.1. In Vivo Antifungal Activity of the Culture Filtrate of the IUM00035 Isolate

**Figure 1:**
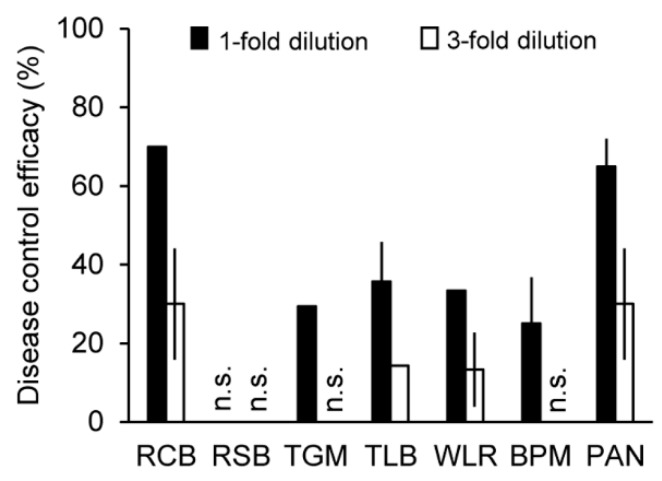
Antifungal activity of the culture filtrate of the IUM00035 isolate. Plant disease control values of the culture filtrate of the IUM00035 isolate. RCB, rice blast; RSB, rice sheath blight; TGM, tomato gray mold; TLB, tomato late blight; WLR, wheat leaf rust; BPM, barley powdery mildew; PAN, pepper anthracnose. n.s. indicates not shown, meaning that disease control effects of the culture filtrate were not observed.

To find a mushroom isolate showing a plant disease control activity, we have examined the in vivo antifungal activity of the culture filtrates of over 100 mushroom isolates against seven plant diseases: rice blast (RCB, caused by *M. oryzae*), rice sheath blight (RSB, caused by *Rhizoctonia solani*), tomato gray mold (TGM, caused by *Botrytis cinerea*), tomato late blight (TLB, caused by *Phytophthora infestans*), barley powdery mildew (BPM, caused by *Blumeria graminis* f. sp. *hordei*), wheat leaf rust (WLR, caused by *Puccinia triticina*), and pepper anthracnose (PAN, caused by *C. coccodes*). Among the culture filtrates of over 100 mushroom isolates, the treatment of undiluted IUM00035 culture filtrate suppressed the development of RCB, TGM, TLB, WLR, BPM, and PAN; however, there was no effect on RSB (Figure 1). In particular, this culture filtrate had strong effects against RCB and PAN with disease control values of 70% and 65% compared with the non-treatment control (distilled water containing 0.025% Tween 20), respectively. Three-fold diluted culture filtrate of the IUM00035 isolate further showed disease suppression of RCB, TLB, WLR, and PAN (Figure 1). Thus, our results suggest that the IUM00035 culture filtrate may contain antifungal substances for the control of plant diseases caused by some plant-pathogenic fungi.

### 2.2. In Vitro Antifungal Activity of the IUM00035 Culture According to Incubation Period

**Figure 2:**
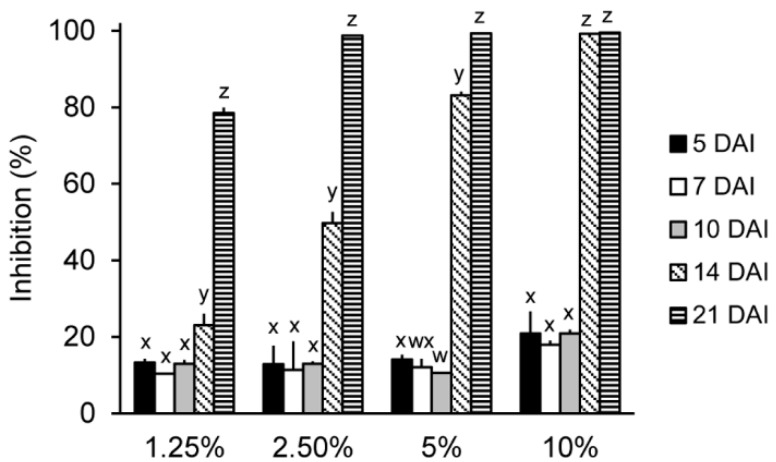
Effects of the incubation time of the IUM00035 isolate on the antifungal activity of its culture filtrate. The IUM00035 culture filtrate was added to wells containing *Saccharomyces cerevisiae* to reach final concentrations of 1.25%, 2.50%, 5%, and 10% (*v*/*v*). The different small letters in each bar indicate a significant difference at *p* < 0.05 (Duncan’s test).

To investigate the antifungal activity of the IUM00035 isolate according to the incubation time, the IUM00035 isolate was incubated for five, seven, 10, 14, and 21 days. For this in vitro assay, the model fungus *Saccharomyces cerevisiae* was used as a target microbe rather than plant-pathogenic fungi such as *M. oryzae* and *C. coccodes.* The reason is that the optical density (OD_600_) was used to calculate the fungal inhibition. When *S. cerevisiae* was treated with 5-, 7-, and 10-day-old culture filtrates of the IUM00035 isolate with different concentrations, the treatments did not show any differences in the antifungal activity regardless of concentrations used. In addition, these culture filtrates exhibited weak antifungal activity ranging from 10% to 20% (Figure 2). However, the 14- and 21-day-old culture filtrates showed strong antifungal activity depending on the concentration used (Figure 2). Similar results have been reported by Imtiaj and Lee (2007) [13] who investigated the antifungal activity of mushrooms according to the culture incubation period and have found that the 20-day-old culture filtrates of *Sterium ostrea*, *Pycnoporus cinnabarinus*, *P. coccineus*, *Oudemansiella mucida* and *Cordyceps sobolifera* had an antifungal activity against plant-pathogenic fungi *B. cinerea*, *C. gloeosporioides* and *C. miyabeanus*. Additionally, Hatvani (2001) noted that culture fluid of *Lentinus edodes* incubated for more than 7 weeks has an antimicrobial activity against *Streptococcus pyogenes*, *Staphylococcus aureus* and *Bacillus megaterium* [7]. Thus, a long incubation time of more than three weeks seems to be required for the production of antifungal substances. Further study will be required to optimize the culture conditions such as the temperature, pH, and light.

### 2.3. Molecular Identification of the IUM00035 Isolate

**Figure 3:**
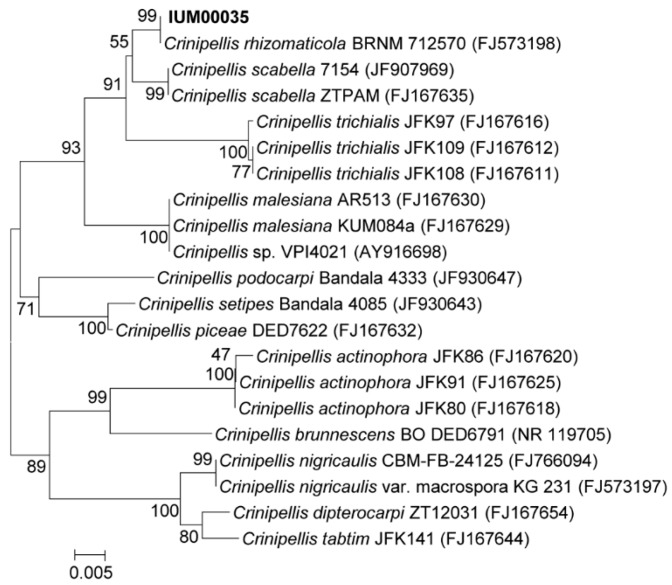
Phylogenetic analysis of the IUM00035 isolate. Phylogenetic analysis was performed based on the ITS region of IUM00035 and the other 20 *Crinipellis* species. The neighbor-joining method was used for this analysis, and numbers at the nodes indicate the levels of bootstrap support (%) by 1000 resampled datasets. NCBI accession numbers of each sequence are in parentheses. Bar, 5 substitution per 1000 nt.

For the molecular identification of the IUM00035 isolate, the internal transcribed spacer (ITS) region was amplified from the IUM00035 gDNA, and the resulting sequences (an amplicon of 616 bp) were searched by the BLASTn program of NCBI (http://www.ncbi.nlm.nih.gov). The ITS region of the IUM00035 isolate had a sequence similarity of 99.8%, 96.4%, and 95.5% to those of *Crinipellis rhizomaticola* BRNM 712570, *C. scabella* 7154, and *C. scabella* ZTPAM, respectively. Comparison of the IUM00035 ITS sequences with 20 *Crinipellis* species revealed that the IUM00035 isolate is phylogenetically close to *C. rhizomaticola* (Figure 3). The genus *Crinipellis* is a mushroom belonging to the order Agricales in Basidiomycota and consists of over 65 species [14]. In particular, *C. rhizomaticola* identified in this study is phylogenetically close to *C. stipitaria*, which produces natural tetraquinane products such as crinipellins [15].

### 2.4. In Vivo Antifungal Activity of the Solvent Extracts of the C. rhizomaticola Culture Filtrate

**Figure 4:**
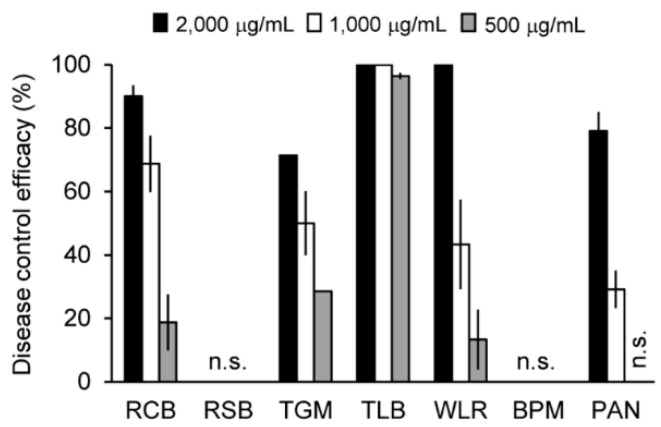
Plant disease control efficacies of the ethyl acetate extract of the *Crinipellis rhizomaticola* IUM00035 culture filtrate. n.s. indicates not shown, meaning that disease control effects of ethyl acetate extracts were not observed. RCB, rice blast; RSB, rice sheath blight; TGM, tomato gray mold; TLB, tomato late blight; WLR, wheat leaf rust; BPM, barley powdery mildew; PAN, pepper anthracnose.

The culture filtrate of the *C. rhizomaticola* IUM00035 isolate was partitioned by organic solvents, and the disease control efficacy of the crude extracts was investigated. The culture filtrate (10 L) was extracted with ethyl acetate and *n*-butanol, successively. Each extract was sprayed at three concentrations of 500, 1000, and 2000 μg/mL onto plants prior to infection with plant-pathogenic fungi. The ethyl acetate extract exhibited an antifungal activity in planta, while the *n*-butanol and water extracts were not active. When the plants were treated with the ethyl acetate extract (2000 µg/mL), the disease control values of TLB, WLR, RCB, TGM, and PAN were 100%, 100%, 90%, 71%, and 79% relative to the non-treatment controls, respectively (Figure 4). Additionally, the in vivo antifungal activity of the ethyl acetate extract was decreased depending on the concentration of the treatment (Figure 4). Of the seven plant diseases, TLB was the most sensitive to the ethyl acetate extract, which resulted in over 96% of the disease control value compared to the non-treatment control, whereas RSB and BPM were not affected by the treatment with ethyl acetate extract (Figure 4).

### 2.5. Isolation and Structure Determination of the Antifungal Compounds

Considering the potent antifungal activity described above, the antifungal compounds were isolated from the ethyl acetate extract by a series of chromatographic procedures. The ethyl acetate extract was subjected to a silica gel column chromatography to yield 10 fractions (E1–E10). The E2 fraction exhibited an antifungal activity in vitro against *M. oryzae* and *P. infestans* (data not shown). Compounds **1** and **2** were finally purified from the E2 fraction by a preparative HPLC system (Figure S1).

**Figure 5:**
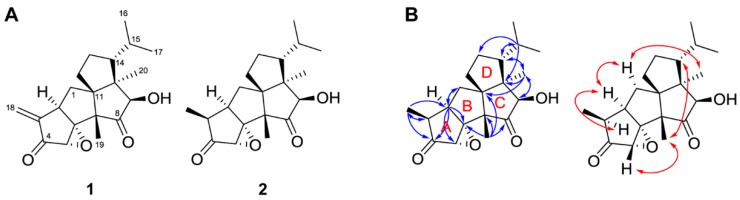
**A**: Structures of **1** and **2**; **B**: Key HMBC (blue arrow) and ROESY (red arrow) correlations of **2**.

The spectroscopic data of **1** and **2** are presented in Table 1 and Table 2, as well as in Supplementary Materials. The ESI-MS spectrum of **1** showed a molecular ion at *m*/*z* 331 [M + H]^+^ (Figure S2). The NMR spectroscopic data and ESIMS data of **1** were in agreement with those of crinipellin A (Figure 5A; Table 1 and Table S1) [16,17,18,19]. The HREIMS of **2** showed a molecular ion at *m*/*z* 332.1985 [M^+^] (Figure S3), which is consistent with the molecular formula C_20_H_28_O_4_ (calculated *m*/*z* 332.1988). The mass difference of 2 amu compared with crinipellin A may indicate the reduction of a double bond in the molecule. Among the known crinipellins, dihydrocrinipellins A and B and crinipellin E have the same molecular formula, i.e., C_20_H_28_O_4_ [18,19,20,21]_._ Based on the comparison of NMR data (Table 1 and Table 2), the structure of **2** was not the same as those of the other known crinipellins [18,19,20,21]. The ^13^C-NMR spectrum showed two ketone carbonyl (*δ*_C_ 212.4 and 216.6), four quaternary sp^3^ (*δ*_C_ 79.4, 61.5, 54.6, and 51.7), six methine sp^3^ (*δ*_C_ 86.1, 58.4, 52.7, 42.0, 40.4, and 29.0), three methylene sp^3^ (*δ*_C_ 33.6, 33.2, and 23.5), and five methyl groups (*δ*_C_ 25.2, 19.8, 16.2, 15.4, and 9.5) (Table 2). Furthermore, the results of the HSQC and HMBC correlations (Figures S9 and S10) suggested that **2** was a diterpene of the crinipellin family (Figure 5B) [17,18,19]. This hypothesis was supported by the presence of the quaternary sp^3^ carbon at *δ*_C_ 61.5 which is shared by all three cyclopentane rings in the tetraquinane scaffold [16,17,18,19]. The proton signal of the Me-18 (*δ*_H_ 0.94) showed HMBC correlations to C-2 (*δ*_C_ 40.4), C-3 (*δ*_C_ 42.0) and C-4 (*δ*_C_ 212.4) (Figure 5B); additionally, correlations from H-2 (*δ*_H_ 2.74) to C-4 (*δ*_C_ 212.4), C-5 (*δ*_C_ 58.4) and C-6 (*δ*_C_ 79.4) revealed that ring A contains a ketone function and an oxirane ring. A comparison of the ^1^H-NMR data (Table 1) of **1** and **2** revealed that the exocyclic methylene group on C-3 in **1** was replaced by a methyl group (Me-18) in **2**. The relative stereochemistry of **2** was obtained from analysis of ROESY spectrum. The ROESY correlations among H-1 (*δ*_H_ 2.18), H-2 (*δ*_H_ 2.74), H-3 (*δ*_H_ 2.45), and Me-20 (*δ*_H_ 1.21) revealed that these protons were on the same side in α-orientation. However, the ROESY correlations among H-5 (*δ*_H_ 3.29), H-14 (*δ*_H_ 1.38), and Me-19 (*δ*_H_ 0.92) indicated that H-5, H-14, and Me-19 were β-oriented (Figure 5B and Figure S11). Thus, **2** was identified as rel-(1a*S*,3*S*,3a*R*,4a*R*,7*R*,7a*R*,8*R*,9a*S*,9b*R*)-8-hydroxy-7-isopropyl-3,7a,9a-trimethylhexahydro-3*H*,5*H*-pentaleno[6a’,1’:5,6]pentaleno[1,6a-*b*]oxirene-2,9(1a*H*,9a*H*)-dione, which was designated as crinipellin I (Figure 5).

Crinipellins are structurally interesting diterpenoids with a unique tetraquinane skeleton and continue to attract the attention of synthetic chemists [16,17]. Crinipellin A, *O*-acetylcrinipellin A, tetrahydrocrinipellin A, crinipellin B, and dihydrocrinipellin B were first reported from *C. stipitaria* [18,20]. Lately, dihydrocrinipellin A, tetrahydrocrinipellin B, and crinipellin C–H have been reported from two different *Crinipellis* strains [19,21]. In the present study, we first isolated crinipellins from *C. rhizomaticola* and identified the structure of crinipellin A and the new derivative crinipellin I.

### 2.6. Minimum Inhibitory Concentrations (MICs) of Crinipellins A and I

The MICs of crinipellin A (**1**) were determined against seven plant-pathogenic fungi: *Alternaria porri*, *B. cinerea*, *C. coccodes*, *Fusarium oxysporum*, *M. oryzae*, *P. infestans*, and *R. solani.* Of the tested fungi, *C. coccodes* was found be the most sensitive to crinipellin A (**1**) (MIC = 1 µg/mL), followed by *M. oryzae*, *P. infestans*, and *B. cinerea* (MICs = 8, 31, and 31 µg/mL) (Table 3). Lorenzen and Anke demonstrated that the high cytotoxic and antimicrobial activities of crinipellins A and B are due to the presence of the electrophilic enone moiety (*α*-methylene-conjugated ketone group) [10]. Other derivatives without the *α*-methylene ketone group such as dihydrocrinipellins A and B, tetrahydrocrinipellins A and B, and crinipellins C and D, were devoid of antimicrobial, antitumor, and anti-inflammatory activities [18,19,21]. Similarly, the *α*-methylene-conjugated ketone was also absent in the structure of crinipellin I (**2**) which could explain its a weak antifungal activity (MICs = 250 μg/mL) against *A. porri*, *C. coccodes*, *M. oryzae*, and *P. capsici*. Additionally, the in vitro antibacterial activity of crinipellins A (**1**) and I (**2**) against nine plant-pathogenic bacteria was also investigated. Crinipellin A (**1**) exclusively exhibited an antibacterial activity against *Acidovorax avenae* subsp. *cattleyae* (MIC = 31 μg/mL), whereas crinipellin I (**2**) had no antibacterial activity (Table S1). Kupka et al. [20] showed that *O*-acetylcrinipellin A, identified from *C. stipitaria*, had an antimicrobial activity against Gram-positive bacteria, such as *Bacillus* spp., suggesting that *O*-acetylcrinipellin A had effects on Gram-positive bacteria but not to Gram-negative strains [20]. In this study, it was found that most Gram-negative plant-pathogenic bacteria were not affected by crinipellin A (**1**), except for *A. avenae* subsp. *cattleyae* (Table S2).

### 2.7. Suppressive Effects of Crinipellin A (1) on Plant Diseases Caused by Plant-Pathogenic Fungi

**Figure 6:**
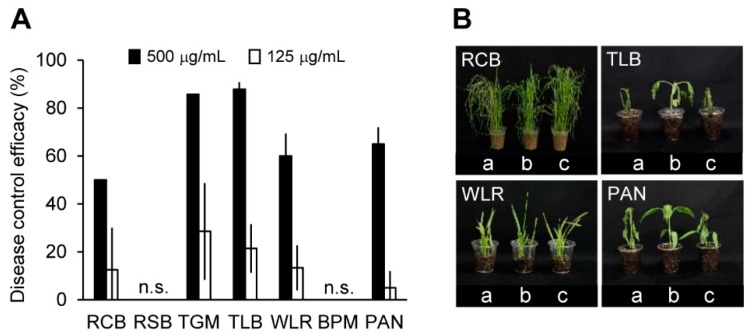
In vivo antifungal activities of crinipellin A (**1**). (**A**) Plant disease inhibitory effects of crinipellin A on plants. RCB, rice blast; RSB, rice sheath blight; TGM, tomato gray mold; TLB, tomato late blight; WLR, wheat leaf rust; BPM, barley powdery mildew; PAN, pepper anthracnose. n.s. indicates not shown, meaning that disease control effects of ethyl acetate extracts were not observed. (**B**) Representatives of the plants treated with crinipellin A (**1**). a, inoculation of pathogens without treatment of crinipellin A; b, treatment with crinipellin A (**1**) (500 µg/mL); c, treatment with crinipellin A (**1**) (125 µg/mL).

To investigate the in vivo antifungal activity of crinipellin A (**1**), plants were treated with 125 and 500 μg/mL of this compound prior to inoculation with the fungal pathogens. As crinipellin I (**2**) was isolated in a very small amount, its in vivo antifungal activity assay was not performed. In greenhouse conditions, crinipellin A (**1**) exhibited an antifungal activity *in plant* against *M. oryzae*, *B. cinerea*, *P. infestans*, *P. triticina*, and *C. coccodes*, which are the causative agents of RCB, TGM, TLB, WLR, and PAN, respectively. The treatment with crinipellin A (**1**) (500 µg/mL) showed disease control values of 88%, 86%, 65%, 60%, and 50%, respectively, against TLB, TGM, PAN, WLR, and RCB, whereas no effect was observed against RSB and BPM (Figure 6). At a concentration of 125 μg/mL, crinipellin A (**1**) had disease control values ranging from 13% to 29% against TGM, TLB, and WLR (Figure 6). In addition to the disease control efficacy, no phytotoxic symptoms were observed in the crinipellin A (**1**)-treated plants (data not shown). The disease control spectrum of crinipellin A (**1**) was similar to that of the IUM00035 culture filtrate and ethyl acetate extract. These results suggested that crinipellin A (**1**) is a major antifungal metabolite produced by the *C. rhizomaticola* IUM00035 isolate and represents potential for development as a natural fungicide to control plant-pathogenic fungi.

## 3. Materials and Methods

### 3.1. General Experimental Procedures

The ESIMS was recorded on a single-quadruple mass spectrometer (Acquity QDa; Waters, Manchester, UK), and the HREIMS was measured by a JMS-700 MStation (JEOL, Tokyo, Japan). The IR spectrum was measured on a Smiths IdentifyIR spectrometer (Danbury, CT, USA) with an attenuated total reflection (ATR) device. The 1D and 2D NMR spectra were recorded by a Bruker Advance 500 MHz spectrometer (Bruker BioSpin, Rheinstetten, Germany) in CD_3_CN or CDCl_3_ (99.8 atom% D, Cambridge Isotope Laboratories, Tewksbury, MA, USA). Chemical shifts were referenced to solvent peaks (*δ*_H_ 1.94 ppm and *δ*_C_ 118.26 ppm for CD_3_CN; *δ*_H_ 7.26 ppm and *δ*_C_ 77.0 ppm for CDCl_3_).

### 3.2. Mushrooms and Culture Conditions

The mushroom isolates used in this study were kindly provided by Dr. Jaehyuk Choi of Incheon National University, and the voucher specimen of the IUM00035 isolate was deposited in the “Culture Collection of Mushrooms” at Incheon National University. The isolates were maintained on potato dextrose agar (PDA) medium at 25 °C. To test the in vivo and in vitro antifungal activity of the mushroom isolates, 10 agar plugs that were punched with an 8 mm-diameter cork borer from culture plates were inoculated into 500 mL of potato dextrose broth (PDB) and incubated at 25 °C for three weeks.

### 3.3. Genomic DNA Extraction and Phylogenetic Analysis

For the isolation of genomic DNA (gDNA), the IUM00035 isolate was grown in 50 mL of PDB medium at 25 °C for 4 days on a rotary shaker (150 rpm), and the gDNA was extracted using the cetyltrimethylammonium bromide (CTAB) procedure as previously described [22]. For phylogenetic analysis, the ITS region was amplified by universal primer sets ITS1 (5′-TCCGTAGGTGAACCTGC GG-3′) and ITS4 (5′-TCCTCCGCTTATTGATATGC-3′). The resulting amplicons were purified using the GeneAll Expin^TM^ PCR purification kit (GeneAll, Seoul, Korea) and then analyzed by a sequencing analysis service (Macrogen, Daejoen, Korea). The resulting sequences were analyzed with the BLASTn program of the NCBI (http://www.ncbi.nlm.nih.gov). Phylogenetic trees were constructed by the neighbor-joining method [23] using the program MEGA 6.0 [24]. The robustness at the individual branching points was estimated by bootstrapping with 1000 replications [25].

### 3.4. Isolation of Antifungal Compounds

The culture broth (10 L) of the IUM00035 isolate was filtered with four layers of cheese cloth, and then successively extracted with ethyl acetate (2 × 10 L), and the aqueous phase was re-extracted with *n*-butanol (2 × 10 L). We obtained 1.4 g of ethyl acetate extract, 1.6 g of *n*-butanol extract, and 19.2 g of water extract. The ethyl acetate extract (1.4 g), which exhibited in vivo antifungal activity, was dissolved in a small amount of chloroform and subsequently loaded onto silica gel column (Merck, Darmstadt, Germany). The column was eluted with mixtures of chloroform/methanol (95.5:0.5, *v*/*v*) to obtain 10 fractions (E1–E10). For the antifungal activity-guided fractionation, all chromatographic fractions were investigated for their antifungal activity against rice blast fungus *M. oryzae*. Compounds **1** (34 mg) and **2** (7 mg) were finally purified from the active fraction E2 (44 mg) with a LC-6AD HPLC system (Shimadzu, Kyoto, Japan) equipped with a Polaris C18-A column (21.2 × 250 mm, 10 μm; Agilent, Santa Clara, CA, USA). The column was eluted with a linear gradient (80–100% for 50 min) of aqueous methanol at a flow rate of 5 mL/min. The effluent was monitored with the SPD-M10Avp photodiode array detector (Shimadzu).

#### 3.4.1. Crinipellin A (**1**)

A colorless solid; UV (MeOH) *λ*_max_ (log*ε*): 235 nm (3.73); ^1^H- and ^13^C-NMR (CDCl_3_) data, Table 1 and Table S1; ESIMS *m*/*z* 331 [M + H]^+^.

#### 3.4.2. Crinipellin I (**2**)

A colorless solid; [*α*]_D_^20^ = −98.2 (*c* = 0.35, MeCN) UV (MeOH): *λ*_max_ (log*ε*): 235 nm (3.70); IR (ATR) *ν*_max_ 2956, 2874, 1735, 1458, 1375 cm^−1^; ^1^H-NMR (CDCl_3_) data, Table 1; ^1^H- and ^13^C-NMR (CD_3_CN) data, Table 2; HREIMS *m*/*z* 332.1985 [M^+^] (calculated for C_20_H_28_O_4_, 332.1988).

### 3.5. Optimization of the Incubation Time for the Production of Antifungal Substances

To investigate the effect of the incubation time on the production of antifungal compounds, IUM00035 was incubated on PDB medium for 5, 7, 10, 14 and 21 days. The antifungal activity of each culture filtrate was measured with the broth microdilution method against *S. cerevisiae*. Culture filtrates of IUM00035 were recovered by centrifugation (20 min; 10,000× *g*) and sterilized with a syringe filter (0.2 μm pore size; Advantec, Tokyo, Japan). Each culture filtrate was diluted 10-, 20-, 40-, and 80-fold with YPD medium, and then yeast cells (OD_600_ = 0.03) were added. The PDB medium was used as a negative control, and azoxystrobin (0.03 µg/mL) was treated as a positive control. After an overnight incubation at 30 °C, OD_600_ values were measured in an XMark microplate spectro-photometer (Bio-Rad, Hercules, CA, USA). The fungal inhibitory effect was calculated with the following equation: inhibition (%) = 100 × [1 − (OD_600_ of treatment/OD_600_ of non-treatment)].

### 3.6. In Vitro Antifungal Activity Assay against Plant Pathogens

The MIC values of crinipellins A (**1**) and I (**2**) against plant-pathogenic fungi were determined by the broth microdilution method using two-fold serial dilutions staring with 250 μg/mL as described by the modified CLSI M38-A method [26]. Spore suspensions (5 × 10^4^ spores/mL) of *A. porri*, *B*. *cinerea, C*. *coccodes*, *F. oxysporum*, *M*. *oryzae*, *P*. *infestans*, and *R. solani* were added to the wells of a 96-well microtiter plate. Crinipellins dissolved in methanol (25 mg/mL) were added and then serially two-fold diluted to reach the final concentrations ranging from 0.5–250 µg/mL. The chemical fungicide azoxystrobin (0.03 µg/mL) and PDB medium containing 1% methanol was used as positive and negative controls, respectively. The microtiter plates were incubated for 2–3 days, and the MIC values were determined by visual inspection of complete growth inhibition [26]. The assay was performed two times with three replicates for each compound at all concentrations investigated.

### 3.7. Disease Control Efficacy Test against Plant Pathogens

To investigate the plant disease control efficacies, solvent extracts (2000, 1000, and 500 µg/mL) and crinipellin A (**1**) (500 and 125 µg/mL) were adjusted by dissolving in a 5% aqueous methanol solution containing 0.025% Tween 20. The final concentration of the methanol in each treatment did not exceed 5% of the volume. As a control, we used 5% aqueous methanol solution containing 0.025% Tween 20. The 1- and 3-fold-diluent culture filtrates containing 0.025% Tween 20 were directly sprayed onto the plants. After treatment with the culture filtrate, solvent extracts, and crinipellin A (**1**), the plants were inoculated with a fungal pathogen and incubated as previously described [27,28]. As hosts for the pathogens, we used rice (*Oryza sativa* L, cv. Nakdong), tomato (*Solanum lycopersicum* cv. Seokwang), barely (*Hordeum sativum* cv. Dongbori), wheat (*Triticum aestivum* cv. Eunpa), and pepper (*Capsicum annuum* cv. Bugang), which were grown in a greenhouse at 25 ± 5 °C for 1–4 weeks. Disease severity was evaluated on days 3 to 11 after inoculation. The experiment was conducted twice with three replicates for each treatment, and the disease control efficacy was calculated with the following equation: control efficacy (%) = 100 × [1 − B/A], where A is the mean lesion area (%) on the leaves or sheaths of the control plants, and B is the mean lesion area (%) on the leaves or sheaths of the treated plants [27,28].

### 3.8. Statistical Analysis

Data were subjected to one-way ANOVA, and the means of the treatments were separated by Duncan’s multiple range test (*p* < 0.05) using the R-software (Version 3.3.1).

## 4. Conclusions

Regardless of the versatile bioactivity of crinipellins such as antitumor, anti-inflammatory, and antimicrobial activities, their antifungal property against plant-pathogenic fungi has not yet been tested. In the present study, crinipellin A (**1**) and a new derivative (crinipellin I (**2**)) were identified in *C. rhizomaticola*, and these compounds exhibited an antifungal activity against plant-pathogenic fungi. These results suggest that *C. rhizomaticola* and crinipellins have potential in the development of natural fungicides for the control of various plant diseases.

## Figures and Tables

**Table 1:** ^1^H-NMR data (500 MHz) of **1** and **2** in CDCl_3_.

Position	1	2
	*δ*_H_, (*J* in Hz)	*δ*_H_, (*J* in Hz)
1	2.51, dd (14.3, 7.5); 1.39, dd (14.2, 13.0)	2.23, dd (14.0, 6.6); 1.20 dd (14.4, 13.7)
2	3.08, dd (12.5, 7.7)	2.69 dt (13.7, 7.0)
3	-	2.55 p (7.3)
5	3.46, s	3.29, s
9	4.40, s	4.42, d (2.7)
12	1.87, dt (9.9, 7.1); 1.59, m	1.88, m; 1.63, m
13	1.59, m	1.63, m
14	1.36, m	1.37, dd (9.3, 2.5)
15	2.09, pd (7.0, 2.8)	2.12, qd (6.8, 2.7)
16	0.85, d (7.0)	0.89, d (6.9)
17	0.82, d (6.8)	0.86, d (6.9)
18	5.48, d (1.4); 6.13, d (1.8)	1.03, d (6.3)
19	1.02, s	1.02, s
20	1.31, s	1.30, s

**Table 2:** ^1^H- and ^13^C-NMR data (500 and 125 MHz) ^a^ of **2** in CD_3_CN.

Position	*δ*_C_, Type	*δ*_H_, (*J* in Hz)	HMBC
1	33.6, CH_2_	2.18, dd (13.9, 6.8); 1.21, m	C-2, 3, 6, 7, 10, 11
2	40.4, CH	2.74, dt (13.9, 7.2)	C-1, 4, 5, 6
3	42.0, CH	2.45, p (7.4)	C-2, 4, 18
4	212.4, CO	-	
5	58.4, CH	3.29, s	C-4, 6
6	79.4, C	-	
7	51.7, C	-	
8	216.6, CO	-	
9	86.1, CH	4.38, d (2.8)	C-8, 10, 20
10	54.6, C		
11	61.5, C		
12	33.2, CH_2_	1.89, ddd (12.3, 8.8, 3.9); 1.59, m	C-7, 10, 11, 13
13	23.5, CH_2_	1.59, m	
14	52.7, CH	1.38, td (9.5, 2.5)	C-9, 10, 15, 16, 17, 20
15	29.0, CH	2.09, pd (6.9, 2.5)	C-10, 13, 14, 16, 17
16	25.2, CH_3_	0.84, d (5.4)	C-14, 15, 17
17	19.8, CH_3_	0.83, d (5.6)	C-16
18	9.5, CH_3_	0.94, d (7.4)	C-2, 3, 4
19	15.4, CH_3_	0.92, s	C-6, 7, 8, 11
20	16.2, CH_3_	1.21, s	C-9, 10, 11, 14

^a^ Assignments (*δ* values) were confirmed by the HSQC and HMBC experiments.

**Table 3:** Minimum inhibitory concentration (MIC) of crinipellins A (**1**) and I (**2**) against plant-pathogenic fungi.

Plant-Pathogenic Fungi	MIC (µg/mL)	
	1	2
*Alternaria porri*	125	250
*Botrytis cinerea*	31	>250
*Colletotrichum coccodes*	1	250
*Fusarium oxysporum*	125	>250
*Magnaporthe oryzae*	8	250
*Phytophthora infestans*	31	250
*Rhizoctonia solani*	125	>250

## Supplementary Materials

The following are available online. Figure S1, HPLC data of compounds **1** and **2**; Figures S2 and S3, MS data for compounds **1** and **2**; Figures S4–S11, NMR spectra of compounds **1** and **2**; Table S1, ^13^C-NMR data (CDCl_3_) of compound **1**; and Table S2, MIC of crinipellins against plant pathogenic bacteria.
