# Supplementary material for: Crinipellins A and I, Two Diterpenoids from the Basidiomycete Fungus Crinipellis rhizomaticola, as Potential Natural Fungicides
Source: Molecules. 2018 Sep 17;23(9):2377. doi: 10.3390/molecules23092377 (PMC6225381; doi:10.3390/molecules23092377)
Supplement: Supplementary file 1 [file molecules-23-02377-s001.pdf]

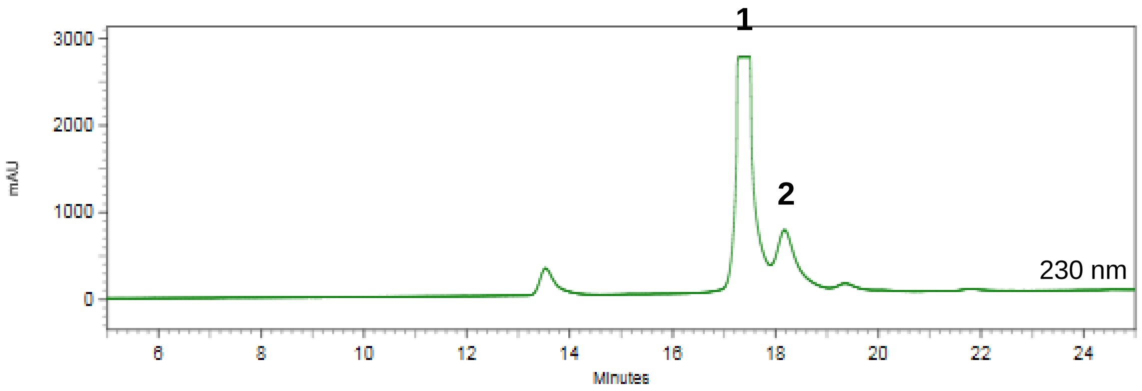

Figure S1. The high performance liquid chromatogram of fraction E2. Compound 1 (34 mg) and 2 (7 mg) were finally purified from the active fraction E2 (44 mg) with a LC-6AD HPLC system (Shimadzu, Kyoto, Japan) equipped with a Polaris C18-A column (21.2 × 250 mm, 10 μm; Agilent, Santa Clara, CA). The column was eluted with a linear gradient (80–100% for 50 min) of aqueous methanol at a flow rate of 5 mL/min. The effluent was monitored with the SPD-M10Avp photodiode array detector (Shimadzu).

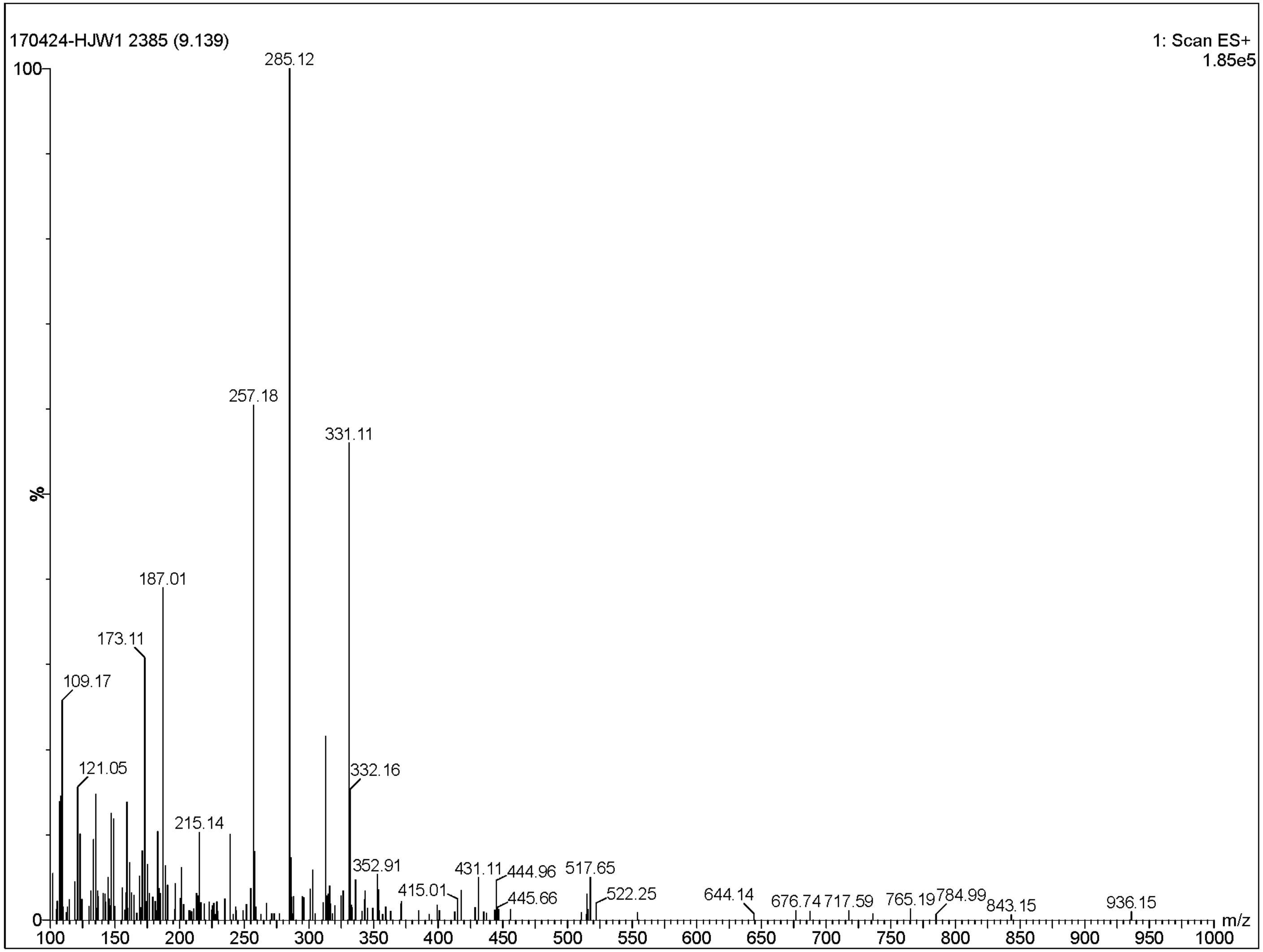

Figure S2. The ESIMS spectrum of compound **1** (m/z 331 $[M + H]^+$)

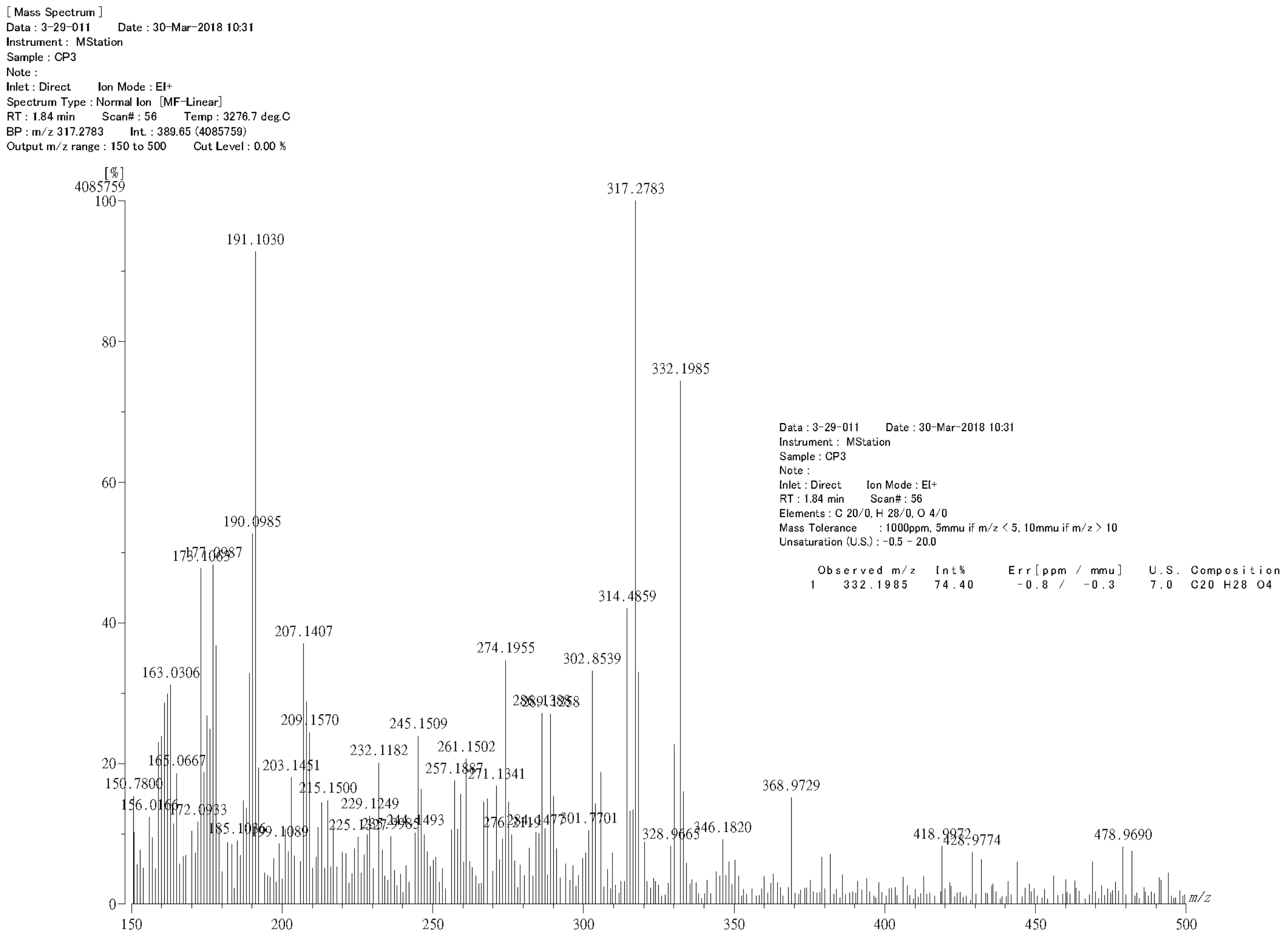

Figure S3. The HREIMS spectrum of compound **2** (observed *m/z* 332.1985 M^+)

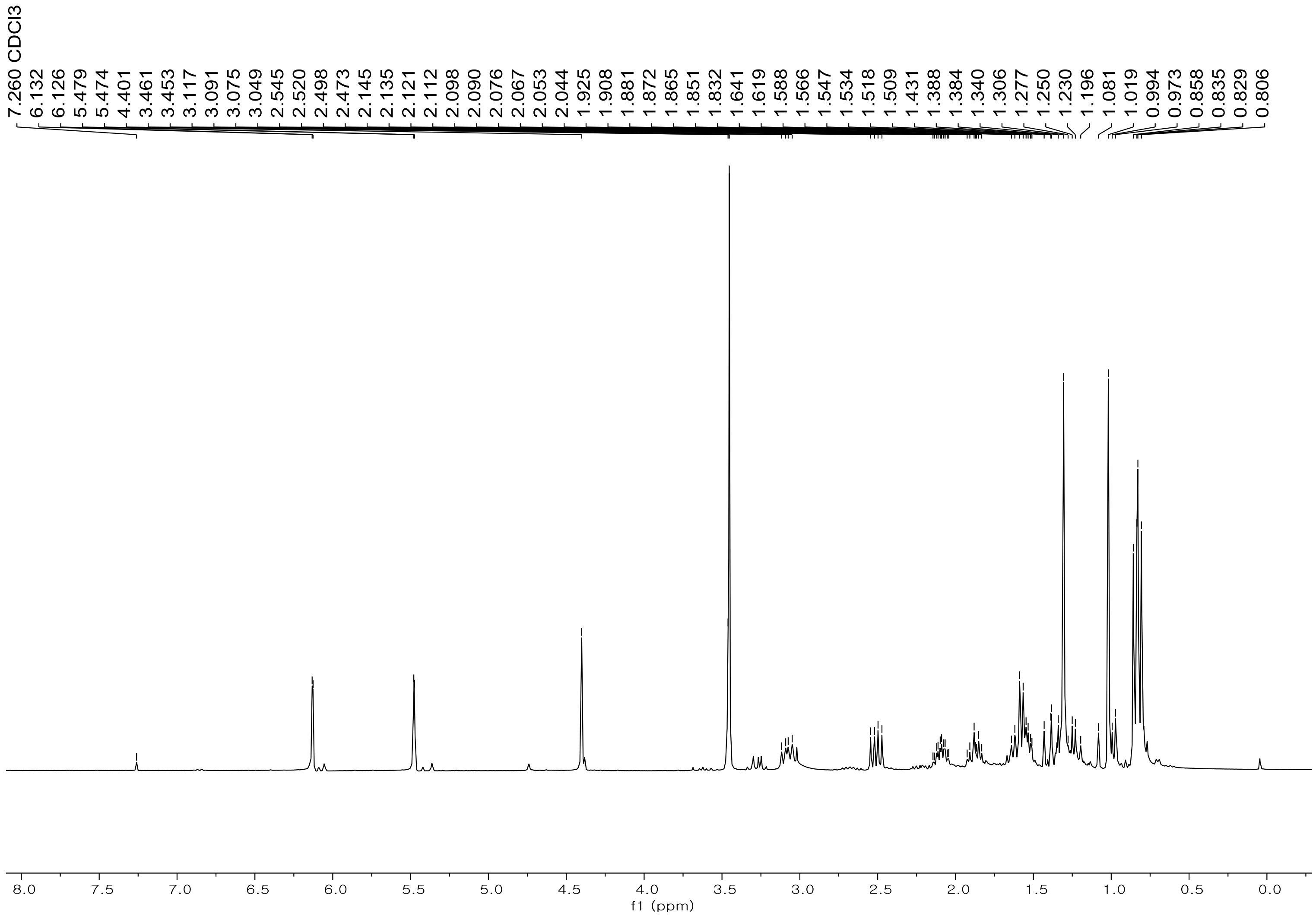

Figure S4. The ^{1}H NMR spectrum of compound **1** (500/125 MHz, $CDCl_3$)

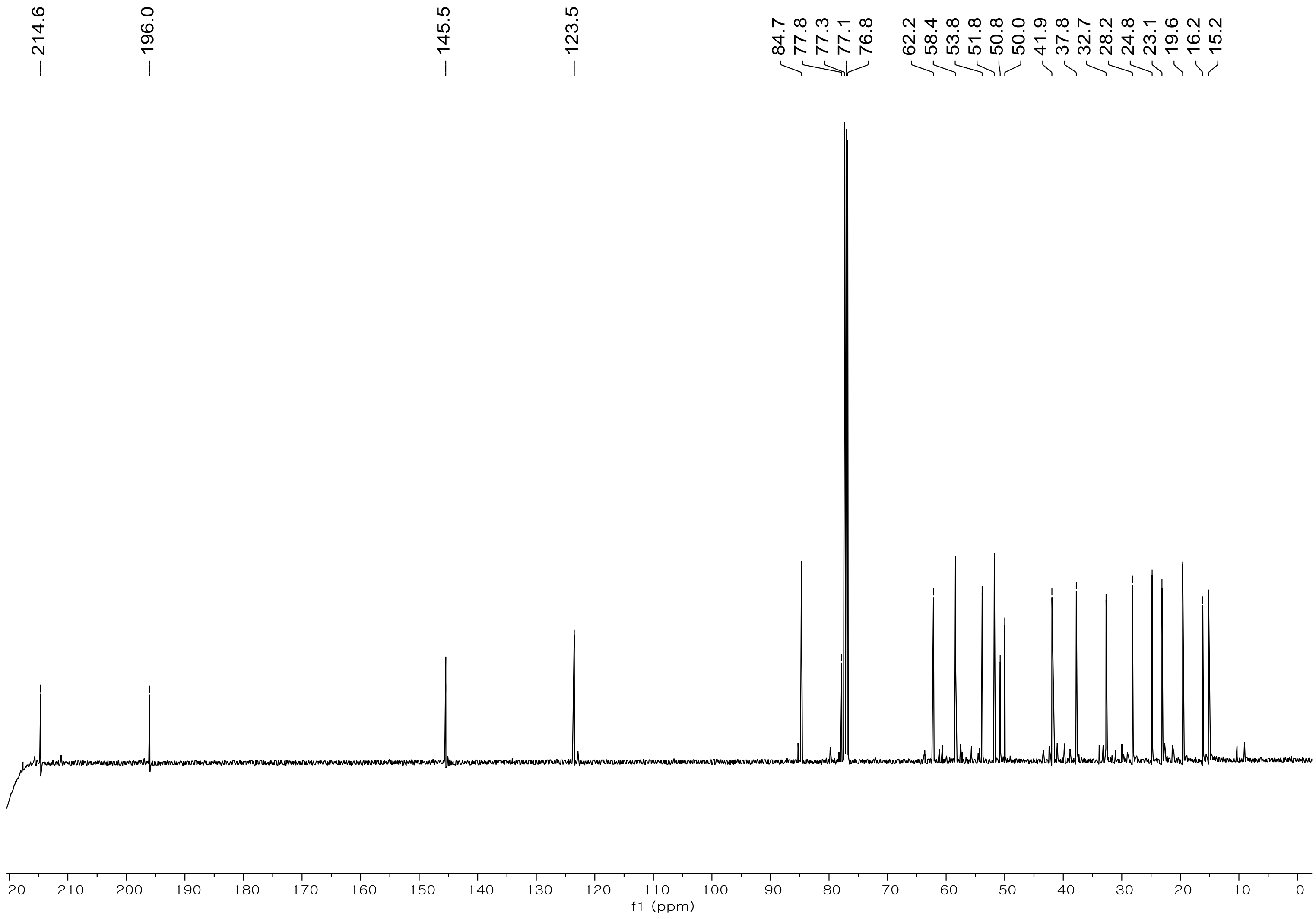

Figure S5. The ^{13}C NMR spectrum of compound **1** (500/125 MHz, $CDCl_3$)

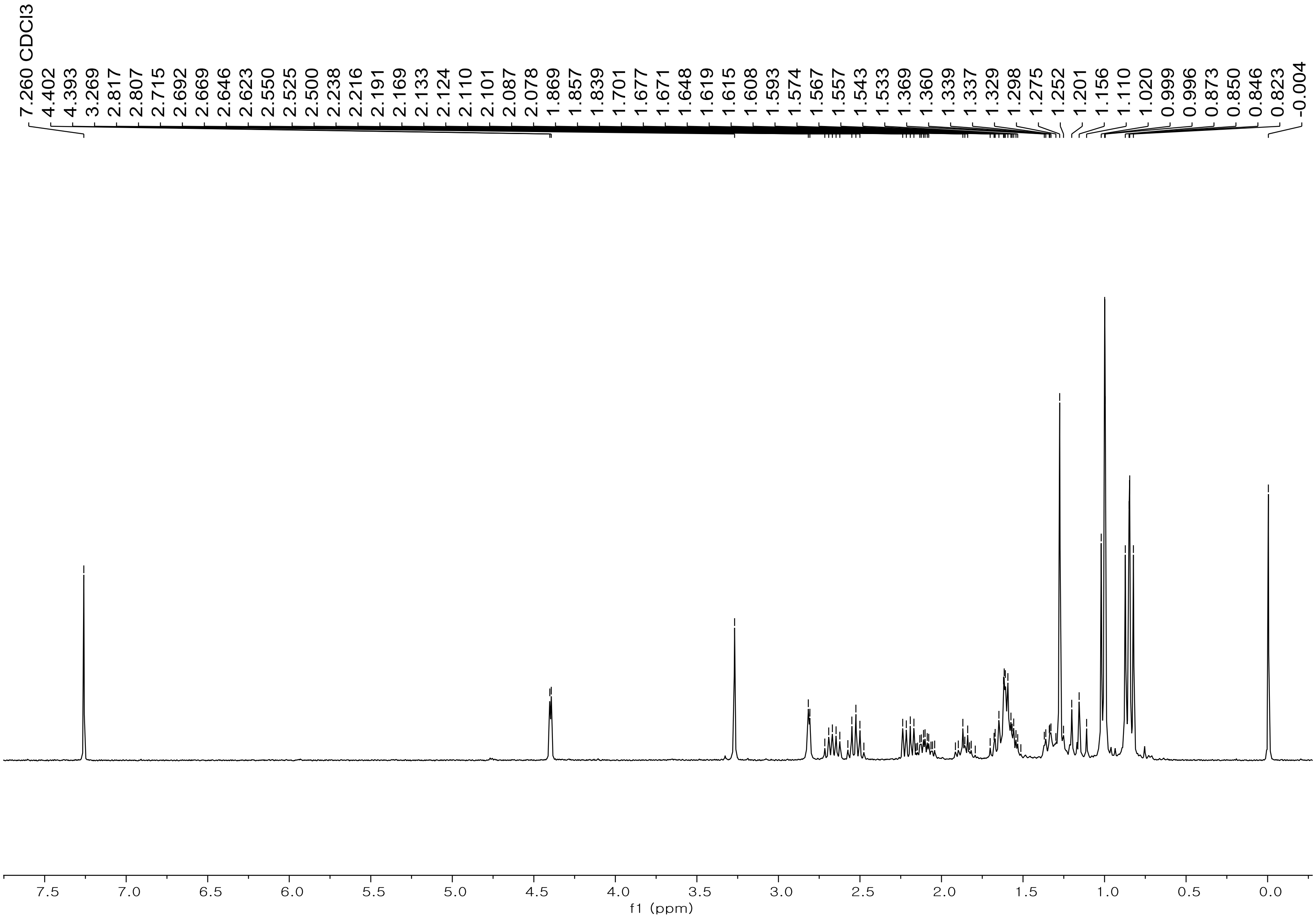

Figure S6. The ^{1}H NMR spectrum of compound **2** (500/125 MHz, $CDCl_3$)

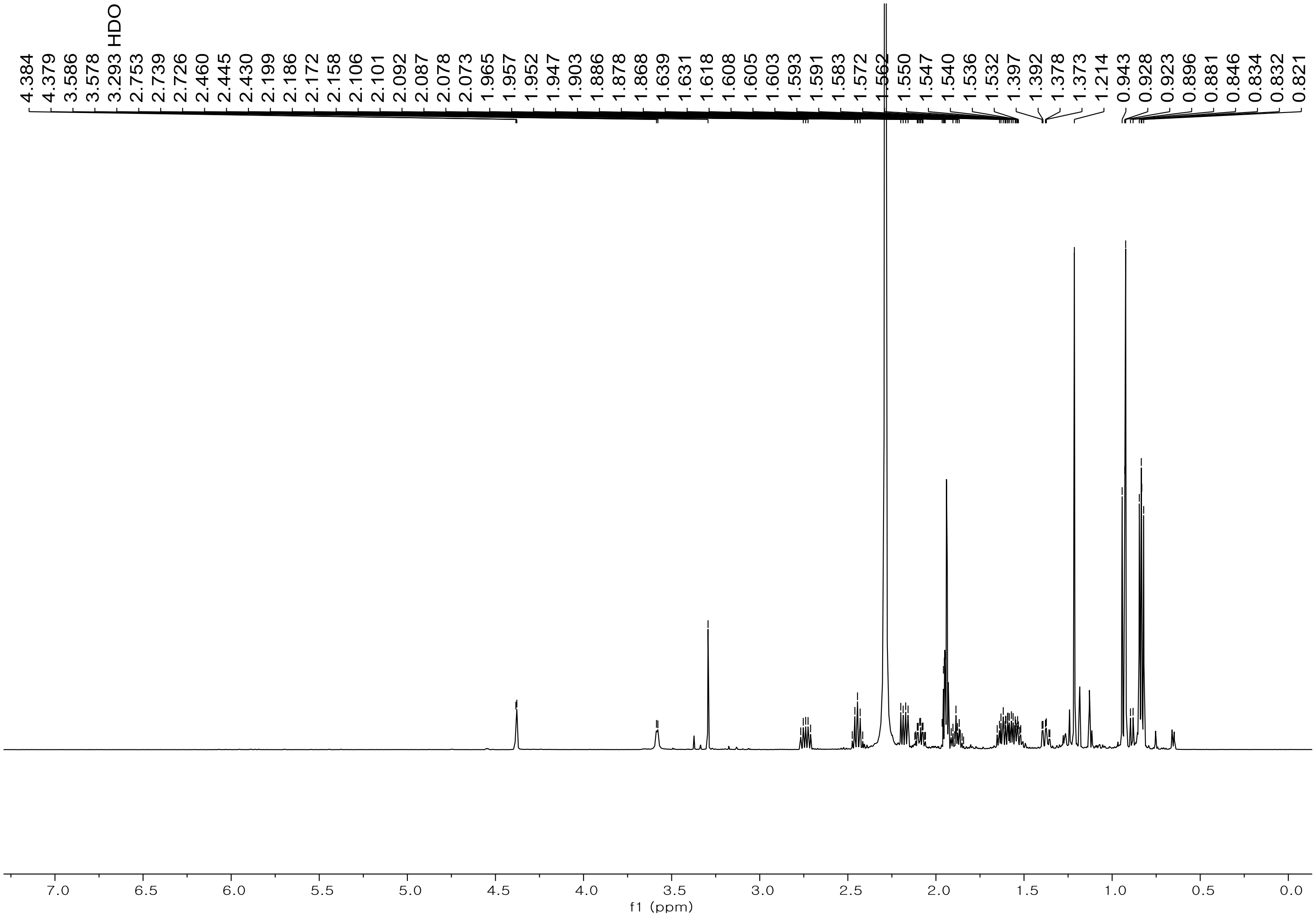

Figure S7. The ^{1}H NMR spectrum of compound **2** (500/125 MHz, CD_3CN)

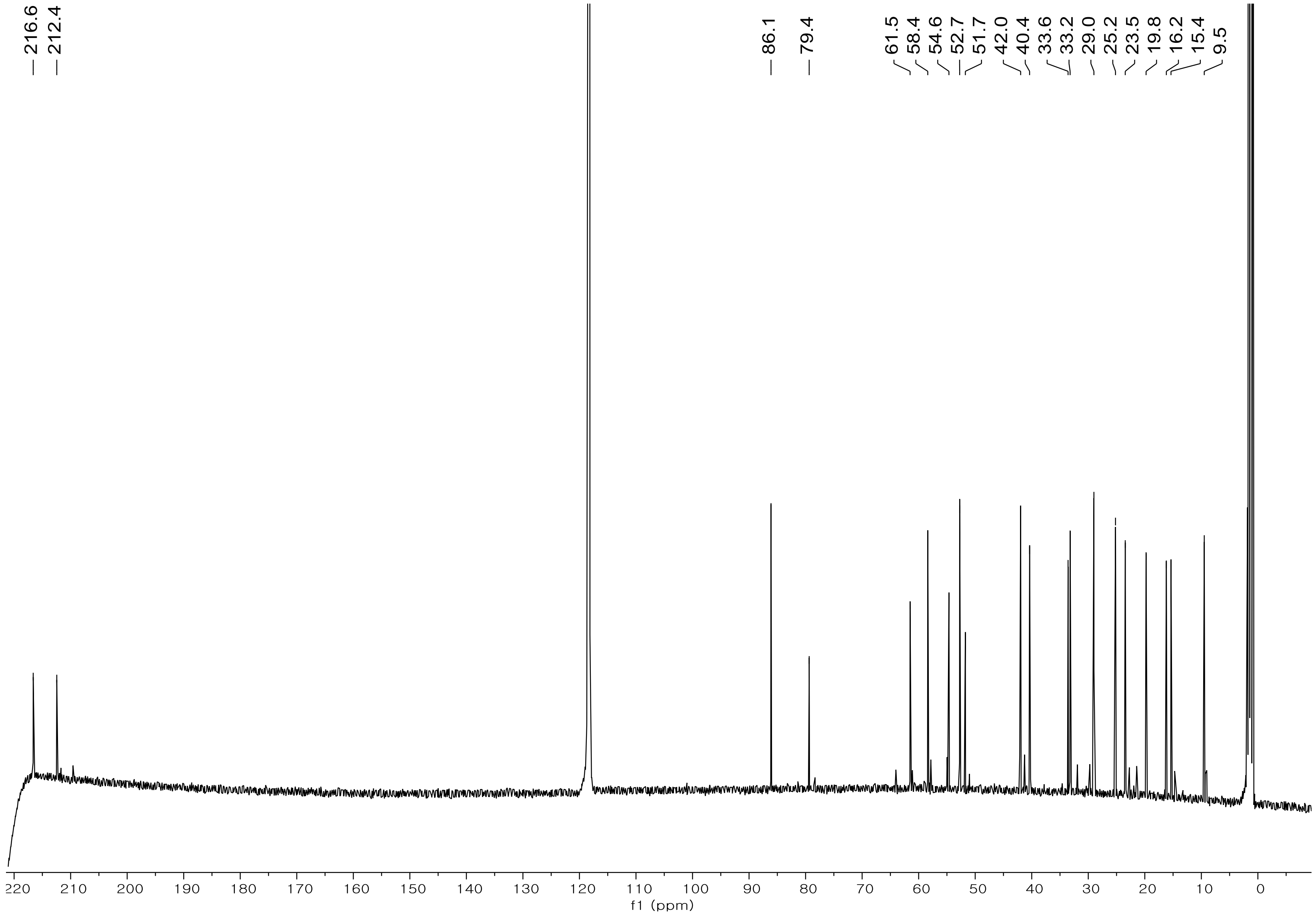

Figure S8. The ^{13}C NMR spectrum of compound **2** (500/125 MHz, CD_3CN)

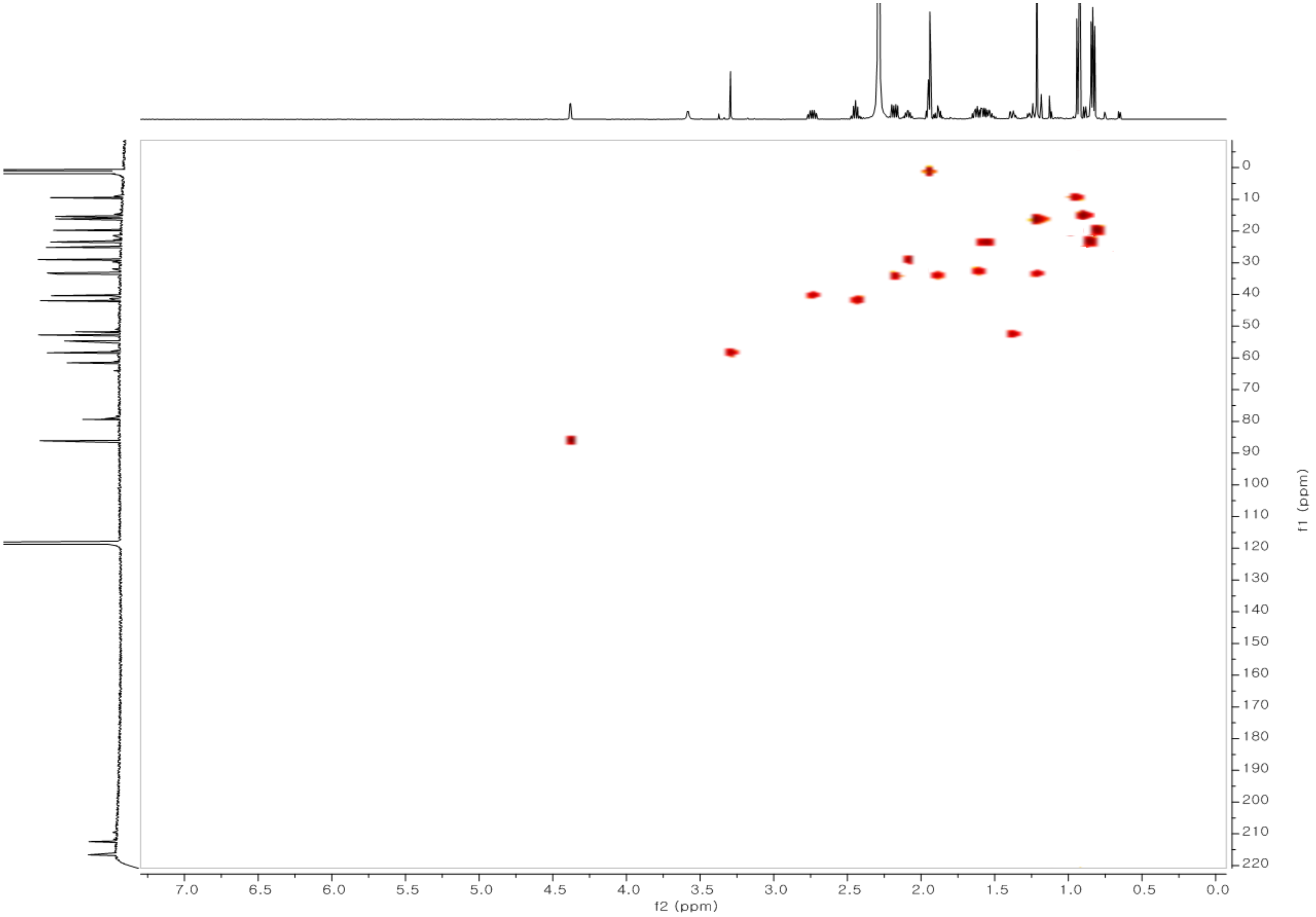

Figure S9. The HSQC spectrum of compound **2** (500/125 MHz, CD_3CN)

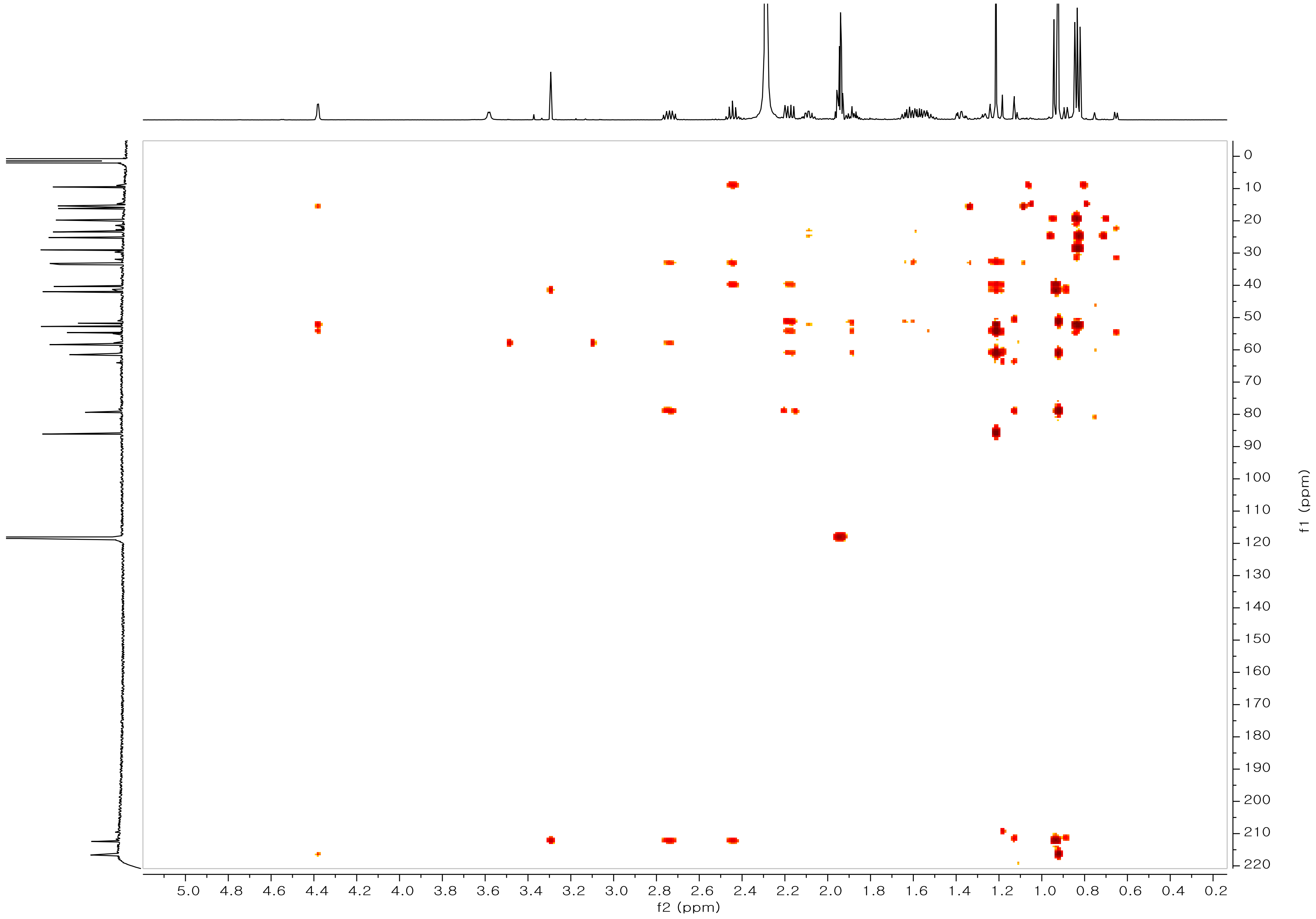

Figure S10. The HMBC spectrum of compound **2** (500/125 MHz, CD_3CN)

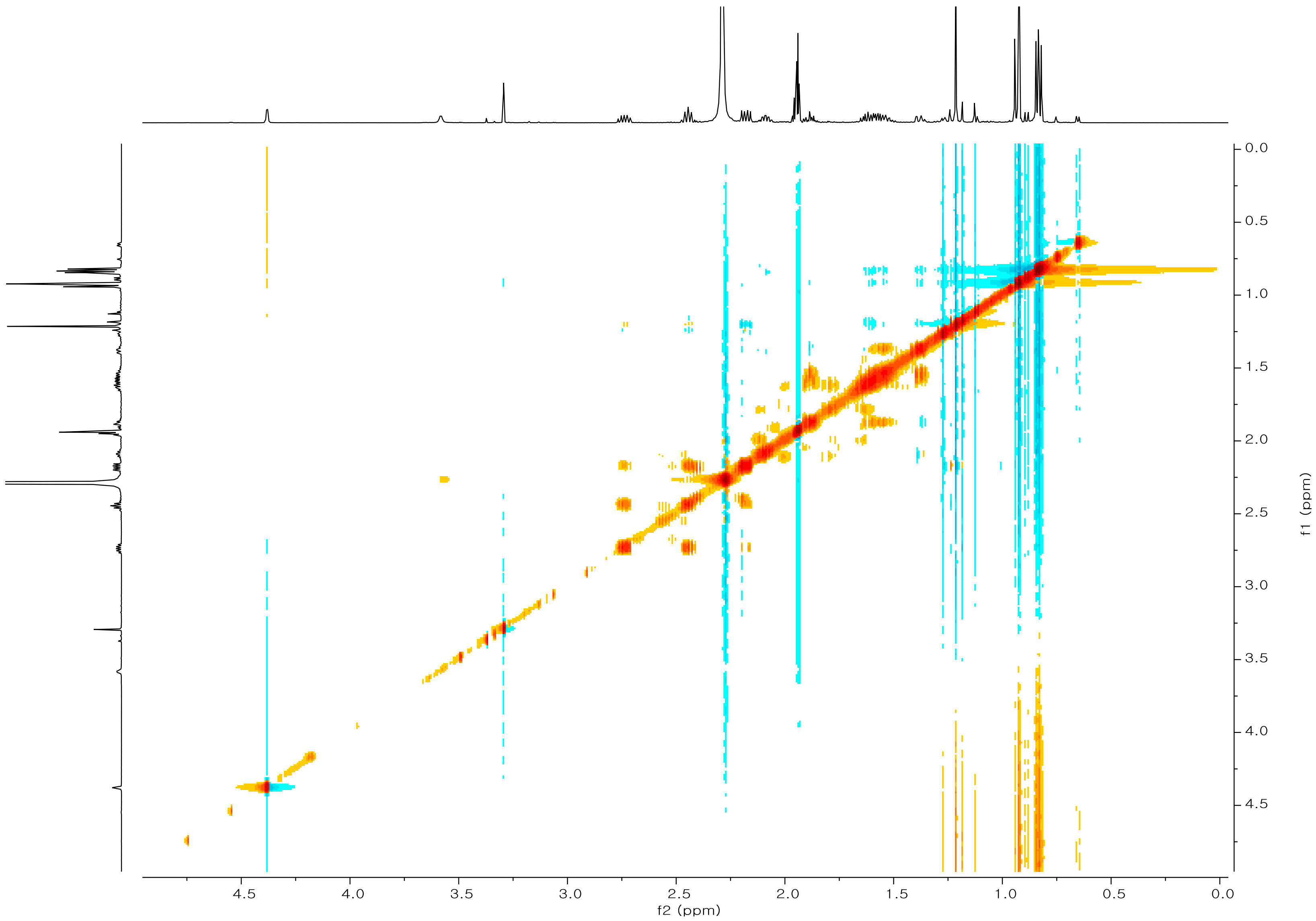

Figure S11. The ROESY spectrum of compound **2** (500 MHz, CD_3CN)

Table S1. The ^{13}C NMR spectroscopic data (δ values in ppm; 500 MHz) for crinipellin A (compound 1) in $CDCl_3$.

Position	δ_C, Type
1	37.8, CH_2
2	41.9, CH
3	145.5, C
4	196.0, CO
5	58.4, CH
6	77.8, C
7	50.0, C
8	214.7, CO
9	84.7, CH
10	53.8, C
11	62.2, C
12	32.7, CH_2
13	23.1, CH_2
14	51.8, CH
15	28.2, CH
16	19.6, CH_3
17	24.8, CH_3
18	123.5, CH_2
19	15.2, CH_3
20	16.2, CH_3

Table S2. Minimum inhibitory concentration (MIC) of crinipellin A (1) and crinipellin I (2) against phytopathogenic bacteria

Phytopathogenic bacteria	MIC (μg/mL)	
	1	2
Acidovorax avenae subsp. *cattleyae*	31	>250
Agrobacterium tumefaciens	>250	>250
Burkholderia glumae	>250	>250
Pectobacterium carotovorum subsp. *carotovorum*	>250	>250
Dickeya chrysanthemi	>250	>250
Pseudomonas syringae pv. *lachrymans*	>250	>250
Xanthomonas arboricola pv. *pruni*	>250	>250
Pseudomonas syringae pv. *actinidiae*	>250	>250
Ralstonia solanacearum	>250	>250

[a] Minimum inhibitory concentration (MIC) values of crinipellins against plant pathogenic bacteria were determined by broth microdilution assay using two-fold serial dilutions starting with 250 μg/mL as described by the modified CLSI M38-A method. Bacteria suspepnsions (1 × 10^4 cells/mL) were used as inocula, tryptic soy broth (BD Biosciences) was used to culture bacteria. Controls containing 1% methanol without the chemical were also included. The microtiter plates were incubated for 2–3 days and MIC was defined as the lowest concentration of crinipellins with no visible bacterial growth.
